# Mortality and its association with chronic alcohol-related diseases in patients admitted to the emergency department for acute alcoholic intoxication: retrospective cohort study

**DOI:** 10.1007/s11739-022-03114-6

**Published:** 2022-10-05

**Authors:** Francesco Palmese, Maria Elena Bonavita, Enrico Pompili, Maria Teresa Migliano, Nicola Reggidori, Cecilia Di Stefano, Marta Grieco, Stefano Colazzo, Manuel Tufoni, Maurizio Baldassarre, Paolo Caraceni, Francesco Giuseppe Foschi, Fabrizio Giostra, Gabriele Farina, Rossella Del Toro, Giorgio Bedogni, Marco Domenicali

**Affiliations:** 1 Department of Medical and Surgical Sciences, Alma Mater Studiorum-University of Bologna, Via Massarenti 9, 40138 Bologna, Italy; 2 Department of Primary Health Care, Internal Medicine Unit Addressed to Frailty and Aging, “S. Maria Delle Croci” Hospital, AUSL Romagna, Ravenna, Italy; 3 Department of Internal Medicine, “Degli Infermi” Hospital, AUSL Romagna, Faenza, Italy; 4 Unit of Semeiotics, Liver and Alcohol-Related Diseases, IRCCS Azienda Ospedaliero-Universitaria of Bologna, Bologna, Italy; 5 Center for Applied Biomedical Research-CRBA, Alma Mater Studiorum-University of Bologna, Bologna, Italy; 6 Emergency Department-Pronto Soccorso, IRCCS Azienda Ospedaliero-Universitaria of Bologna, Bologna, Italy

**Keywords:** Epidemiology, Mortality, Alcohol use disorder, Alcohol-induced disorders, Emergency department

## Abstract

We assessed long-term mortality and its association with chronic alcohol-related diseases in patients admitted to the emergency department (ED) because of acute alcoholic intoxication (AAI). A retrospective cohort study was performed at the ED of Sant’Orsola-Malpighi Hospital, Bologna, Italy. 3304 patients, corresponding to 6415 admissions for AAI, who accessed the ED from January 1, 2005, to December 31, 2017, were studied. The ED electronic registry system was used to assess living status on 08 May 2020 and to obtain the prespecified potential predictors, i.e., age at first admission, sex, alcohol use disorder (AUD), substance use disorder (SUD), more than 1 admission to ED for trauma, mental and behavioral disorders, neurological disorders, and cardiovascular disease. The median follow-up time was 9.3 years and the time on risk was 30,053 person years (PY) with a death rate corresponding to 4.42 (95% CI 3.74–5.26) per 1000 PY (*n* = 133 deaths). The death rate was higher in patients with AUD (17.30) than in those without AUD (1.98) and in those with SUD (13.58) than in those without SUD (3.80). Lastly, there was a clearly higher death rate among AUD+ SUD+ (20.89) compared to AUD–SUD–patients (1.74). At multivariable Cox regression, AUD, SUD, and liver cirrhosis were strong and independent predictors of time-to-death. Using standardized mortality ratios, a clear excess of mortality was evident for all the age bands from (40–45] to (60–65] years. Mortality is higher in AAI than in the general population and chronic alcohol-related diseases are strongly associated with it.

**Supplementary Information:**

The online version contains supplementary material available at 10.1007/s11739-022-03114-6.

## Introduction

Alcohol-related disease is a major cause of mortality, disability, and social disruption worldwide [1]. Alcohol use disorder (AUD) is a chronic relapsing disease characterized by compulsive alcohol consumption associated with loss of control and psychological distress [2].

AUD is underdiagnosed in most countries. In Italy, a recent report by the National Observatory on Alcohol shows that, out of 710,000 estimated harmful users of alcohol in need of treatment, only 72,000 are followed by the alcohol services of the National Health System [3]. Thus, 90% of alcohol addicts in Italy cannot access treatment because it is not requested either by the addicted person or by a health professional in charge of identifying the problem.

Early recognition of AUD is crucial because chronic alcohol abuse leads to long-term disease, e.g., liver cirrhosis, cardiovascular disease, and neurological disease. In Italy, the Emergency Department (ED) plays a central role in identifying patients with AUD, who often come there because of acute alcoholic intoxication (AAI) [3–6].

There are presently no data on long-term mortality in patients with AAI admitted to the ED and all the available studies of short-term mortality of AAI performed in the ED have focused on acute consequences of alcohol abuse, e.g., major trauma [7].

The aim of the present retrospective cohort study was therefore to assess long-term mortality and its association with chronic alcohol-related diseases in patients with AAI admitted to the ED of a large metropolitan hospital.

## Material and methods

The study is reported following the STROBE guidelines [8].

### Study design and setting

The present retrospective cohort study enrolled consecutive patients admitted for AAI to the ED of the Sant’Orsola-Malpighi Hospital (Bologna, Italy) from January 1, 2005, to December 31, 2017. The study was approved by the Ethical Committee of the Emilia-Romagna Region (CE-AVEC, number of approval 302/2020/Oss/AOUBo). Written informed consent was waived in view of the retrospective nature of the study.

### Participants

Patients with AAI were eligible for the present study. We operationally defined AAI as at least one of the following diagnoses as coded by the International Classification of Diseases version 9 or 10 depending on the study period: (1) alcohol abuse; (2) alcohol dependence syndrome; (3) toxic effect of alcohol; (4) accidental poisoning by and exposure to alcohol; (5) intentional self-poisoning by and exposure to alcohol; (6) alcohol poisoning; (7) evidence of alcohol involvement determined by blood alcohol level. The only exclusion criterion was age < 12 years.

### Variables and data sources

#### Outcome variable

The outcome variable was mortality rate. The last ascertainment of living status was made on 08 May 2020 using the ED electronic registry system, which is linked to the registry offices of the municipality where the patients were living at the time of death or at the end of the study.

#### Predictor variables

The prespecified predictors of mortality, obtained from the ED electronic registry system using ICD-9 or ICD-10 codes depending on the study period, were: (1) age at first (“baseline”) admission (years); (2) male sex (0 = no; 1 = yes); (3) AUD (0 = no; 1 = yes); (4) substance use disorder (SUD) (0 = no; 1 = yes); (5) more than 1 admission to ED for trauma (0 = no; 1 = yes); (6) mental and behavioral disorders (0 = no; 1 = yes); (7) neurological disorders including non-cardiac syncope, vertigo, dizziness, and tremors (0 = no; 1 = yes); (8) cirrhosis (0 = no; 1 = yes); (9) cardiovascular disease (0 = no; 1 = yes).

### Sample size

No formal calculation of sample size was performed.

### Missing data

There were no missing data.

### Statistical analysis

Continuous variables are reported as median (50th percentile) and interquartile range (IQR, 25th and 75th percentiles) and discrete variables as the number and proportion of subjects with the characteristic of interest. Death rates were calculated per 1000 person years (PY). In addition to the overall death rate, we calculated the death rate associated with AUD, SUD, and their combination. A prespecified multivariable Cox regression model was used to evaluate the association between survival time and potential predictors. Survival time was calculated as the difference between the present and the previous admission or death or censoring, employing multiple records per patient [9]. Such format allowed to easily code for time-varying covariates. The time-fixed covariates of the Cox regression model were sex (discrete: 1 = male; 0 = female) and baseline age (continuous, years) divided by 5, to represent an increment of 5 years. The time-varying covariates of the Cox model were AUD (discrete, 0 = no; 1 = yes), SUD (discrete, 0 = no; 1 = yes), more than 1 admission to ED for trauma (discrete, 0 = no; 1 = yes), mental and behavioral disorders (discrete, 0 = no; 1 = yes), neurological disorders (discrete, 0 = no; 1 = yes), liver cirrhosis (discrete, 0 = no; 1 = yes), and cardiovascular disease (discrete, 0 = no; 1 = yes). Fractional polynomials were used to test whether the relationship between continuous age and survival time was linear given the other discrete covariates [10]. The relationship was found to be linear and modeled as such. The 95% confidence intervals of the model coefficients and of the corresponding hazard ratios were calculated using the bootstrap on 1000 random samples with replacement [11]. The proportional hazards assumption made by the Cox model was ascertained using Schoenfeld residuals [12]. The Akaike information criterion (AIC) and the Bayesian information criterion (BIC) were used to assess model fit [13]. Standardized mortality ratios (SMR) were calculated every five years of age using reference data from Northern Italy for the years 2011–2017 (http://dati.istat.it/Index.aspx). Confidence intervals for rates and SMR were calculated using the quadratic approximation to the Poisson log likelihood for the log-rate parameter [14]. Statistical analysis was performed using Stata 17.0 (Stata Corporation, College Station, TX, USA).

## Results

### Participants

During the 13 years of the study, 883,683 emergency admissions were performed at the ED of the Sant’Orsola-Malpighi Hospital. Among these, 6415 (0.7%) were admissions for AAI that involved 3304 single patients. 133 of these 3304 patients died between 17 Sep 2007 and 08 May 2020.

### Baseline measurements

The baseline measurements of the patients are given in Table 1.

**Table 1:** Demographic and clinical characteristics at the first (“baseline”) admission of 3304 patients admitted to the Emergency Department for acute alcoholic intoxication between January 1, 2005, and December 31, 2017

	*N* = 3304
Italian citizen	2068 (62.6%)
Male sex	2195 (66.4%)
Age (years)	30 (22; 43)
Aged < 18 years	172 (5.2%)
Homeless	262 (7.9%)
Alcohol use disorder	357 (10.8%)
Substance use disorder	175 (5.3%)
More than 1 admission to ED for trauma	425 (12.9%)
Mental and behavioral disorders	477 (14.4%)
Neurological disorders	257 (7.8%)
Dementia	12 (0.4%)
Cirrhosis	63 (1.9%)
Cardiovascular disease	138 (4.2%)

63% of the patients were Italian citizens and 66% were males. The median (IQR) age was 30 (22; 43) years and 5% of the patients were aged < 18 years. 8% of patients were homeless; 11% had already received a diagnosis of AUD and 5% one of SUD; 13% had previously had more than 1 admission to ED for trauma; 14% had mental and behavioral disorders and 8% neurological disorders, while dementia was uncommon (0.4%); cirrhosis was present in 2% and cardiovascular disease in 4% of the patients.

### Measurements at the last available admission

Table 2 compares the characteristics at the last available admission of the 133 patients who died during the follow-up with those of the 3171 patients who were alive at the end of the follow-up.

**Table 2:** Demographic and clinical characteristics at the last available admission of 3304 patients admitted to the Emergency Department for acute alcoholic intoxication between January 1, 2005, and December 31, 2017, stratified by living status as determined on May 8, 2020

	Alive *N* = 3171	Dead *N* = 133
Italian citizen	1950 (61.5%)	118 (88.7%)
Male sex	2089 (65.9%)	106 (79.7%)
Age (years)	29 (22; 43)	61 (50; 73)
Aged < 18 years	172 (5.4%)	0 (0.0%)
Homeless	273 (8.6%)	24 (18.0%)
Alcohol use disorder	461 (14.5%)	83 (62.4%)
Substance use disorder	190 (6.0%)	26 (19.5%)
More than 1 admission to ED for trauma	557 (17.6%)	75 (56.4%)
Mental and behavioral disorders	518 (16.3%)	47 (35.3%)
Neurological disorders	267 (8.4%)	44 (33.1%)
Dementia	10 (0.3%)	8 (6.0%)
Cirrhosis	69 (2.2%)	29 (21.8%)
Cardiovascular disease	119 (3.8%)	41 (30.8%)

The patients who died were mostly Italian citizens (89%) and males (80%) and had a median age much higher than that of those who were still alive (61 vs. 29 years). AUD (62 vs. 14%), SUD (19 vs. 6%), more than 1 admission to ED for trauma (56 vs. 18%), mental and behavioral disorders (35 vs. 16%), neurological disorders (33 vs. 8%), cirrhosis (22 vs. 2%) and cardiovascular disease (31 vs. 4%) were also commoner among the patients who died.

#### Follow-up

The median number of admissions per patient was 1, but it was extremely skewed, ranging from 1 to 200. The median follow-up time was 9.3 years (range: 3 days to 15.3 years) and the time on risk was 30,053 PY with a death rate corresponding to 4.42 (95% CI 3.74 to 5.26) per 1000 PY (*n* = 133 deaths).

### Death rate associated with AUD, SUD, and their combination

Table 3 reports the death rate in patients with AUD, SUD, and their combination.

**Table 3:** Death rate in patients with AUD, SUD, and their combination

		Death	PY	Rate (95% CI)
AUD–	–	50	25.25	1.98 (1.51–2.65)
AUD+	–	83	4.80	17.30 (14.04–21.52)
SUD–	–	107	28.14	3.80 (3.16–4.62)
SUD+	–	26	1.91	13.58 (9.36–20.37)
AUD–	SUD–	42	24.20	1.74 (1.29–2.38)
AUD–	SUD+	8	1.05	7.60 (3.88–16.87)
AUD+	SUD–	65	3.94	16.51 (13.05–21.16)
AUD+	SUD+	18	0.86	20.89 (13.35–34.22)

Death rate is calculated as death/person-time (per 1000)

*AUD* alcoholic use disorder, *SUD* substance use disorder, *PY* person years

The death rate was higher in patients with AUD (17.30 per 1000 PY) than in those without AUD (1.98 per 1000 PY), and in patients with SUD (13.58 per 1000 PY) than in those without SUD (3.80 per 1000 PY). Lastly, there was a clearly higher death rate among AUD+ SUD+ (20.89) compared to AUD- SUD-patients (1.74).

### Multivariable prediction of time-to-death

Model 1 of Table 4 is the prespecified Cox regression model used to investigate the relationship between time-to-death and its potential predictors.

**Table 4:** Multivariable Cox regression of time-to-death

	Model 1	Model 2	Model 3
Male	1.64* [1.05–2.56]	1.61* [1.01–2.57]	1.68* [1.07–2.64]
Baseline age/5 (years)	1.48*** [1.39–1.58]	1.49*** [1.39–1.58]	1.48*** [1.40–1.58]
Alcohol use disorder	2.13*** [1.36–3.34]	2.62*** [1.72–4.00]	–
Substance use disorder	3.69*** [2.13 to 6.41]	4.22*** [2.47–7.21]	4.00*** [2.25–7.11]
More than 1 admission to ED for trauma	2.02** [1.31–3.12]	–	2.46*** [1.66–3.64]
Mental and behavioral disorders	0.96 [0.62–1.47]	0.98 [0.64–1.51]	1.10 [0.71–1.72]
Neurological disorders	1.46 [0.98–2.20]	1.49 [0.98–2.28]	1.62* [1.06–2.46]
Liver cirrhosis	2.54*** [1.58–4.08]	2.73*** [1.71–4.37]	3.07*** [1.87–5.05]
Cardiovascular disease	1.50 [0.94–2.40]	1.62* [1.03–2.55]	1.55 [0.96–2.50]
*AIC*	1645	1657	1657
*BIC*	1706	1711	1711

Values are hazard ratios with 95% bootstrapped confidence intervals in brackets (see Statistical analysis for details)

*AIC* akaike information criterion, *BIC* Bayesian information criterion

*p < 0.05, **p < 0.01, ***p < 0.001

The multivariable predictors of time-to-death identified by Model 1 were male sex (HR = 1.64), baseline age (HR = 1.48 for an increase of 5 years), AUD (HR = 2.13), SUD (HR = 3.69), more than 1 admission to ED for trauma (HR = 2.02), and liver cirrhosis (HR = 2.54).

Because AUD and more than 1 admission for trauma were strongly associated (Spearman's rho = 0.52, *p* < 0.0001), suggesting multicollinearity [10], we fitted a second multivariable model without the more than 1 admission for trauma predictor (Model 2), and a third multivariable model without the AUD predictor (Model 3). Although there was some loss of gain in model fit for Models 3 and 2 as compared to Model 1 as detected by both AIC and BIC, the point estimate of the HR and the lower 95% CI of the AUD predictor increased from Model 1 to Model 2 and the same was true for the more than one hospitalization for trauma predictor from Model 1 to Model 3. Because this may be a sign of multicollinearity, we preferred to employ Model 2, including AUD alone, for further analysis.

It should be noted that the removal of the more than 1 admission to ED for trauma predictor from Model 1 allowed cardiovascular disease to be statistically significant in Model 2 and neurological disorders to be statistically significant in Model 3. This is not clinically relevant, at least in the present cohort, because of the imprecision of the estimate of the corresponding HR as shown by the wide 95% CI. Incidentally, the same is true for male gender in all models.

**Fig. 1:**
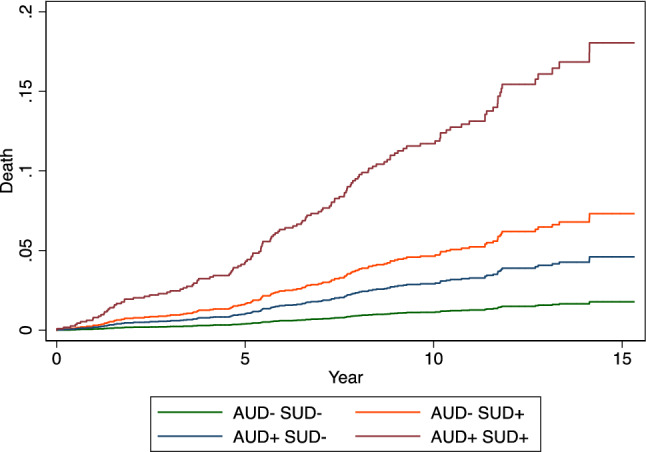
Failure function of death estimated by Model 2 of Table 3 for AUD and SUD with all other variables set to their mean value

Figure 1 plots the failure function of death estimated by Model 2 of Table 3 for AUD and SUD with all other variables set to their mean value, showing that SUD adds to AUD as the probability of death is concerned, confirming, and extending the findings obtained at univariable and bivariable analysis (Table 3).

### Standardized mortality ratios

Table 5 gives the SMR for the study cohort. A clear excess of observed vs. expected deaths can be detected from 40 to 65 years and for the (75–80] years age band.

**Table 5:** Standardized mortality ratios

Age band (years)	PY	Obs. deaths	Exp. deaths	SMR (95% CI)
(15–20]	765	0	0.15	–
(20–25]	3685	0	1.11	–
(25–30]	5161	2	1.55	1.29 (0.32–5.17)
(30–35]	4392	0	1.32	–
(35–40]	3421	4	1.71	2.34 (0.88–6.23)
(40–45]	2871	**8**	2.30	**3.48 (1.74–6.97)**
(45–50]	2560	**9**	3.64	**2.47 (1.29–4.75)**
(50–55]	2114	**12**	4.86	**2.47 (1.40–4.35)**
(55–60]	1571	**17**	5.97	**2.85 (1.77–4.58)**
(60–65]	1193	**17**	7.99	**2.13 (1.32–3.42)**
(65–70]	832	10	9.15	1.09 (0.59–2.03)
(70–75]	572	11	10.76	1.02 (0.57–1.85)
(75–80]	334	**18**	11.16	**1.61 (1.02 to 2.56)**
(80–85]	250	10	15.25	0.66 (0.35 to 1.22)
(85–90]	187	11	22.30	0.49 (0.27 to 0.89)
> 90	105	4	23.24	0.17 (0.06 to 0.46)
Total	30,053	133	122.44	1.09 (0.92 to 1.29)

Excess deaths and corresponding SMR are given in bold

*PY* person years, *Obs.* observed, *Exp*. expected, *SMR* standardized mortality ratio, *95% CI* 95% confidence interval

There were no deaths among patients aged ≤ 25 years, whose characteristics are given in Supplementary Table 1. This ancillary analysis was performed because binge drinking represents the first cause of death in subjects ≤ 25 years [3, 15, 16]. Patients aged ≤ 25 years were mostly Italian citizens (69% vs. 58%) with a lower frequency of homeless patients (2% vs. 11%) as compared to those aged > 25 years. The median (IQR) hour of the ED admission of the younger patients was 3:00 (2:00; 5:00) while that of the older patients was 10:00 (03:00; 20:00). Interestingly, 2% of the younger patients had already received a diagnosis of AUD and 2% one of SUD. Supplementary Fig. 1 plots the proportion of patients aged ≤ 25 years admitted to the ED during the study period. In line with the available literature [3, 16], the figure shows an increase in the proportion of patients aged ≤ 25 years starting from 2010.

## Discussion

The present retrospective cohort study was aimed at assessing long-term mortality and its association with chronic alcohol-related diseases in patients with AAI admitted to the ED. Our estimate of the frequency of admissions for AAI over the study period (0.7%) is in line with national data (≤ 1%) [3]. We quantified the mortality rate in 4.42 (95% CI 3.74–5.26) per 1000 PY and identified AUD, SUD, and liver cirrhosis as strong and independent predictors of time-to-death. Importantly, there was a clear excess of deaths, as detected by the standardized mortality ratio (SMR), for the age bands from (40–45] to (60–65] years and in the age band (75–80] years.

The main strength of the present study is that it is the first one to investigate long-term mortality and its association with chronic alcohol-related diseases in patients with AAI. We performed such study in the ED because in Italy and most countries it is the first interface between the National Health System and persons with undiagnosed AUD. Another strength is the sample size, consisting of 3304 patients for a total of 6415 admissions performed during 13 consecutive years. A third strength is the fact that the median (IQR) age at the baseline admission was only 30 (22; 43) making it less likely at the population level that death occurs because of chronic disease related to alcohol consumption.

The present study has several limitations. We obtained mortality data from the registry offices of the municipality where the patients were living at the time of death or at the end of the study. It is thus possible that some deaths might have gone undetected among homeless patients. Nonetheless, 18% of the deaths which were recorded by the registry offices occurred in homeless patients. Another limitation is that it is likely that there is a proportion of cases where the ICD coding system was inappropriately used, leading to misclassification of AAI and of the predictors of interest. Having not performed an internal cross-validation of the ICD coding in this cohort, we cannot tell how much large such proportion is. Moreover, as the predictors are concerned, although it would be very useful to stratify them by etiology, e.g., viral vs. alcoholic liver cirrhosis, this cannot be reliably done from the ED database, as it is generally true for death certificates [14]. Another limitation is that this is a single-center study performed in a hospital which is not a hub center for major trauma. Thus, we may have missed major traumas related to acute alcohol abuse even if the corresponding deaths—if any—were available in the ED system. Moreover, the aim of the present study was to evaluate the association between mortality and chronic alcohol-related diseases.

AAI is often an underestimated cause of access to the ED which can hide serious underlying chronic diseases [17]. It is well known that chronic diseases such as liver cirrhosis, AUD [18], and SUD [19, 20] carry a mortality rate higher than that of the general population. Mild AAI requires a short stay in the ED and only supportive therapy and does not lead to serious complications [4]. In most cases, however, AAI underlies AUD, and recognizing this association may lead to immediate care and prevention of chronic alcohol-related diseases [21].

Our study shows for the first time that the mortality rate of a large population accessing the ED for AAI is associated not only with the acute effects of alcohol but also with chronic alcohol-related diseases. This suggests that the ED is a crucial place not only for treating the acute effects of AAI but also to identify patients with AUD and send them to the alcohol services of the National Health System. The most interesting finding of the present study is how SUD adds to AUD as the mortality rate is concerned. This fact is expected from the available literature [22, 23], but this is the first time that this relationship is reported for long-term mortality and for patients with AAI seen at the ED and has important clinical and organizational implications. Interestingly, while there were no deaths among the patients aged ≤ 25 years, 2% of them had already received a diagnosis of AUD and 2% one of SUD, which is not negligible.

## Conclusion

Mortality is higher in AAI than in the general population and chronic alcohol-related diseases are strongly associated with it.

## Supplementary Information

Below is the link to the electronic supplementary material.

Supplementary file1 (DOCX 66 KB)

Supplementary file2 (DOCX 15 KB)

## Data Availability

Anonymized data may be made available upon reasonable request.
