# Supplementary material for: Mortality and its association with chronic alcohol-related diseases in patients admitted to the emergency department for acute alcoholic intoxication: retrospective cohort study
Source: Intern Emerg Med. 2022 Oct 5;18(1):257–63. doi: 10.1007/s11739-022-03114-6 (PMC9883355; doi:10.1007/s11739-022-03114-6)
Supplement: Supplementary file 1 — Supplementary file1 (DOCX 66 KB) [file 11739_2022_3114_MOESM1_ESM.docx]

**Supplementary figure 1** – Proportion of patients aged ≤ 25 years admitted to the emergency department during the study period. Values are marginal proportions and cluster 95% confidence intervals estimated by using logistic regression with year of hospitalization as discrete predictor.
