# Supplementary material for: Mortality and its association with chronic alcohol-related diseases in patients admitted to the emergency department for acute alcoholic intoxication: retrospective cohort study
Source: Intern Emerg Med. 2022 Oct 5;18(1):257–63. doi: 10.1007/s11739-022-03114-6 (PMC9883355; doi:10.1007/s11739-022-03114-6)
Supplement: Supplementary file 2 — Supplementary file2 (DOCX 15 KB) [file 11739_2022_3114_MOESM2_ESM.docx]

**Supplementary Table 1 –** Demographic and clinical characteristics at the first (“baseline”) admission of 3304 patients admitted to the Emergency Department for acute alcoholic intoxication between January 1, 2005, and December 31, 2017, stratified by age.

|  | > 25 years | ≤ 25 years |
| --- | --- | --- |
|  | *N*=2099 | *N*=1205 |
| Hour at admission | 10 (3; 20) | 3 (2; 5) |
| Italian citizen | 1,233 (58.7%) | 835 (69.3%) |
| Male sex | 1,496 (71.3%) | 699 (58.0%) |
| Age (years) | 39 (31; 50) | 21 (19; 23) |
| Homeless | 239 (11.4%) | 23 (1.9%) |
| Alcohol use disorder | 335 (16.0%) | 23 (1.9%) |
| Substance use disorder | 145 (6.9%) | 30 (2.5%) |
| More than 1 admission for trauma | 358 (17.1%) | 67 (5.6%) |
| Mental and behavioural disorders | 414 (19.7%) | 64 (5.3%) |
| Neurological disorders | 221 (10.5%) | 35 (2.9%) |
| Dementia | 12 (0.6%) | 0 (0.0%) |
| Cirrhosis | 63 (3.0%) | 0 (0.0%) |
| Cardiovascular disease | 130 (6.2%) | 8 (0.7%) |
